# Salud pública y salud mental: hacia una historia (descentrada) de la epidemiología psiquiátrica

**DOI:** 10.1590/S0104-59702025000100013

**Published:** 2025-04-07

**Authors:** Rafael Huertas

**Affiliations:** i Instituto de Historia, Consejo Superior de Investigaciones Científicas (Espanha). Madrid – España, orcid.org/0000-0002-4543-7180, rafael.huertas@csic.es

**Figure:**
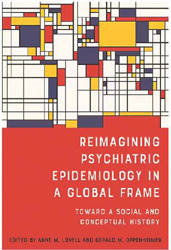
LOVELL, Anne M.; OPPENHEIMER, Gerald M. (ed.). *Reimagining psychiatric epidemiology in a global frame: toward a social and conceptual history*. Rochester, NY: University of Rochester Press and Suffolk, 2022. 340p.

La historia de las epidemias tiene una gran tradición en el ámbito de la historia de la medicina o de la salud. La peste, el cólera, la fiebre amarilla, la gripe, por poner solo algunos ejemplos de los muchos existentes, han sido objeto de muy diversos estudios que han analizado aspectos biológicos (descripciones clínicas, debates epidemiológicos etc.), respuestas políticas, repercusiones económicas y reacciones socioculturales ante eventos epidémicos o pandémicos del pasado. Un interés historiográfico que aumentó ante nuevas e inquietantes enfermedades, como el sida en los años 1980 o la covid-19 más recientemente. Asimismo, la llamada transición epidemiológica dio lugar a una creciente preocupación por las enfermedades crónicas, como las cardiovasculares o el cáncer y, en los últimos tiempos, por los problemas de salud mental. Sin embargo, el libro que reseñamos (Lovell, Oppenheimer, 2022) se sale de esta tradición y nos propone, en primer lugar, una clara distinción entre la historia de las enfermedades epidémicas (infecciosas o no) y la historia de la epidemiología como disciplina y herramienta fundamental de la salud pública en una triple vertiente: metodológica, social e institucional.

En segundo lugar, aborda una historia de la epidemiología psiquiátrica, poniendo de manifiesto sus peculiaridades con relación al desarrollo histórico de la epidemiología de las enfermedades físicas. Finalmente, en tercer lugar, plantea con acierto la necesidad de superar el eurocentrismo o americanocentrismo (del Norte) y el racismo epistemológico, con trabajos que abordan estudios de caso en África, Asia y América Latina. Una suerte de “historia descentrada” (Davis, 2011) aplicada aquí a la historia de la salud.

La tesis fundamental que se defiende en *Reimagining psychiatric epidemiology in a global frame* es que el proceso de construcción disciplinar de la epidemiología psiquiátrica no ha sido uniforme, ni ha estado sustentado en paradigmas comunes, como sucedió posiblemente con la epidemiología de las enfermedades cardiovasculares o del cáncer, sino que existe una pluralidad de epidemiologías psiquiátricas impulsadas por un amplio abanico de elementos: aspectos científicos, estrategias políticas y económicas, ideales reformistas, culturas nacionales, influencias internacionales y objetivos de control social. Considerados en conjunto, los distintos capítulos de este libro describen un desarrollo desigual de las epidemiologías y conceptos psiquiátricos en distintos contextos socioculturales, pero todas influidas por una circulación transnacional y selectiva de conceptos, técnicas y conocimientos, que se movían a través de caminos multidireccionales entre y dentro del norte y del sur globales.

Los editores de la obra, la antropóloga social Anne M. Lovell y el historiador Gerald M. Oppenheimer han diseñado el libro en tres bloques. El primero, titulado “Constructing mental health utopias and dystopias with epidemiology”, contiene tres capítulos. En primer lugar, el historiador Rhodri Hayward explora el desarrollo de la epidemiología psiquiátrica en Gran Bretaña en el marco de la utopía que supuso el llamado Estado del bienestar tras la Segunda Guerra Mundial. Una época de reformas sociales con áreas de justicia redistributiva, como la seguridad social, que, sin estar exentas de contradicciones, se mantuvieron hasta que, todo hay que decirlo, las crisis económicas y las políticas neoliberales posteriores hicieron añicos la utopía socialdemócrata del bienestar. En segundo lugar, la antropóloga japonesa Junko Kitanaka analiza desde una novedosa perspectiva el famoso *Hisayama study,* un estudio de cohorte prospectivo diseñado para evaluar los factores de riesgo de enfermedades relacionadas con el estilo de vida, como el accidente cerebrovascular, la enfermedad coronaria, la hipertensión, la diabetes y la demencia, en población japonesa. El componente de demencia, que es el que se estudia aquí, sirvió como modelo de una “autovigilancia participativa” que generó tanto resistencias ante posibles escenarios distópicos (invasión de privacidad, estigma, control social etc.) como posibilidades de desarrollo comunitario y de utopías basadas en los avances de la biomedicina. Finalmente, el psiquiatra y epidemiólogo Naomar Almeida-Filho centra su aportación en la epidemiología psiquiátrica brasileña, a la que considera una “formación histórica regional” que solo puede explicarse en relación con luchas políticas, sociales e institucionales específicas, en marcado contraste con la epidemiología dominante del Norte global. Un discurso situado en el ámbito ideológico de la salud colectiva, al que el autor no es, ni mucho menos, ajeno.

El segundo bloque, “Troubling the boundaries of psychiatric epidemiology”, contiene dos artículos que se aventuran en las fronteras metodológicas de la epidemiología. El historiador Gerald Oppenheimer y el epidemiólogo Richard Neugebauer analizan de qué manera los componentes emocionales y de salud mental se incorporaron en EEUU a los factores de riesgo de enfermedades crónicas, en particular de las cardiovasculares, ampliando de esta manera los límites científicos y el papel de la epidemiología psiquiátrica a la comprensión de la enfermedad “somática”. Por su parte, el historiador de la psicología Emmanuel Delille analiza los primeros estudios epidemiológicos de la sección de psiquiatría transcultural de la Universidad McGill en Canadá, destacando la labor de Eric Wittkower y sus esfuerzos por diseñar y poner en marcha una amplia red internacional para obtener datos sobre trastornos mentales en poblaciones de diversos continentes.

Los estudios culturales pusieron de manifiesto la necesidad de “descentrar el lugar de la enunciación” (Harding, 1998) para tener en cuenta los saberes y prácticas generados desde los grupos subalternos (mujeres, colonizados, obreros, enfermos etc.) y, por supuesto, locas y locos. De manera similar, el tercer bloque del libro que comentamos, “Decentering psychiatric epidemiology in a postcolonial world”, propone superar el lugar central que tradicionalmente ha ocupado la epidemiología psiquiátrica europea y norteamericana para estudiar el conocimiento y las metodologías que han surgido en el Sur global. Así, el historiador Matthew Heaton describe el desarrollo de la epidemiología psiquiátrica en la Nigeria poscolonial; Anne Lovell analiza el papel desempeñado en Senegal por el médico militar francés Henri Collomb, fundador de la escuela Fann de etnopsiquiatría, un tema que ha sido objeto de trabajos simultáneos al que nos ocupa (Ndoye et al., 2022); Pratap Sharan y sus colaboradores sitúan el nacimiento de la epidemiología psiquiátrica en la India en el contexto de la colonización británica y llaman la atención sobre las dificultades de los epidemiólogos indios para analizar e intervenir sobre las realidades de los grupos subalternos de la India poscolonial debido a la influencia y el “mimetismo” de la epidemiología y la psiquiatría occidental. Finalmente, Harry Yi-Jui Wu, médico e historiador de la medicina, analiza el desarrollo de la epidemiología psiquiátrica en Taiwán bajo la colonización japonesa, destacando una serie de rasgos específicos y diferenciadores que se fueron matizando tras la independencia y defendiendo la existencia de una epidemiología psiquiátrica intrínseca y propia del extremo oriente, de manera similar a lo que Almeida-Filho define para Brasil como epistemes (o formaciones históricas) regionales.

En suma, *Reimagining psychiatric epidemiology in a global frame* es un libro colectivo que ofrece un acercamiento a la epidemiología psiquiátrica como disciplina científica y académica desde la historia social y conceptual. El diálogo entre profesionales de la historia, la antropología, la medicina y la epidemiología resulta fructífero, y la selección de los estudios de caso apropiada y sugerente para establecer comparaciones, pero también conexiones, así como para poner en valor las epistemologías regionales. Una historia crítica, y descentrada, que permite reflexionar sobre aspectos científicos, éticos y de políticas públicas y proporcionar elementos que contribuyan a explorar alternativas políticas. Pero también una posible respuesta, clara y contundente, a la repetida pregunta de Gayatry Spivak (1988): ¿puede hablar el subalterno?
